# Evaluation of an optimal agar medium for detecting hypervirulent Klebsiella pneumoniae using string test

**DOI:** 10.1099/acmi.0.000834.v3

**Published:** 2024-09-20

**Authors:** Naoki Watanabe, Akari Masuda, Tomohisa Watari, Yoshihito Otsuka, Kazufumi Yamagata, Miyuki Fujioka

**Affiliations:** 1 Clinical Laboratory, Kameda Medical Center, Higashi-cho 929, Kamogawa, Chiba 296-8602, Japan; 2 Graduate School of Health Sciences, Hirosaki University, Hon-cho 66-1, Hirosaki, Aomori, 036-8564, Japan

**Keywords:** agar media, diagnostic accuracy, hypervirulent *Klebsiella pneumoniae*, string test

## Abstract

The string test is a screening method for detecting hypervirulent *Klebsiella pneumoniae* (hvKp). Agar media are used for string tests; however, the effect of the type of media on the test results remains unclear. We aimed to determine the optimal agar medium and cutoff value for the string test. We performed the string test on 99 *Klebsiella* strains using different agar media: sheep blood, chocolate, Drigalski’s and MacConkey. The diagnostic accuracy was calculated in concordance with the *rmpA*, *rmpA2* or *iucA* gene levels. The diagnostic accuracy rates for sheep blood, chocolate, Drigalski’s and MacConkey agar were 0.79, 0.75, 0.73 and 0.64, respectively. When the cutoff was changed from 5 to 10 mm, the diagnostic accuracy rate for sheep blood agar decreased from 0.79 to 0.65. Our findings suggest that the type of agar medium impacts the string test results and sheep blood agar with a 5-mm cutoff is the optimal condition for detecting hvKp.

## Data Summary

An example of the string test is shown in Supplemental Video 1. The strains and test data are presented in Tables S1 and S2. These files are available on Figshare (https://doi.org/10.6084/m9.figshare.26106493.v1[1]).

## Introduction

*Klebsiella pneumoniae* is an important member of the *Enterobacteriaceae* family and a significant human pathogen. It is classified into classical *K. pneumoniae* (cKp) and hypervirulent *K. pneumoniae* (hvKp) based on distinct characteristics [2]. hvKp has a higher possibility of causing liver abscess, sepsis and invasive infection than cKp [3]. Additionally, this pathogen is associated with the development of endophthalmitis [4]. Identifying hvKp is crucial for estimating infection sites and guiding treatment; therefore, hvKp screening is important in clinical practice.

Screening methods for hvKp include the detection of specific virulence genes and the utilization of the string test [5]. Previous studies have suggested that the *rmpA*, *rmpA2* and *iucA* genes are beneficial markers for hvKp and have an accuracy of more than 95% [6]. The string test, as described by Fang *et al*. [5], assesses hypermucoviscosity, a distinctive phenotype often associated with hvKp. Fang *et al*. used 5 % sheep blood agar for the string test and defined a positive result as the presence of 5-mm strings [5]. Hypervirulence and hypermucoviscosity are different phenomena [7]. However, the string test is used as a complementary test because it is easy to perform without any reagents or equipment.

Several previous studies have used agar media that are different from 5 % sheep blood agar for string tests [7,10]. Additionally, several studies have defined a 10-mm string as an indication of a positive result [11,12]. Nevertheless, the impact of media type and cutoff on the string test results remains unclear. The optimization of agar media and cutoff value may improve the performance of the string test in detecting hvKp. Therefore, we collected string test-positive and -negative clinical isolates and used these strains to evaluate the effect of agar medium type and cutoff value on the accuracy of the string test in hvKp diagnosis. Compared with the string test, the *rmpA*, *rmpA2* and *iucA* genes can identify hvKp with high accuracy. Therefore, hvKp was defined by *rmpA*, *rmpA2* and *iucA* genes in this study. The string test was performed using four types of agar media, and their diagnostic performance and appropriate cutoff values for hvKp diagnosis were evaluated.

## Methods

This study was conducted at Kameda Medical Center in Japan from November 2022 to August 2023. We isolated *K. pneumoniae* strains and performed string tests using various agar media and cutoffs. Subsequently, we calculated the diagnostic accuracy, sensitivity and specificity of the string tests for hvKp detection.

### Strain collection

**Fig. 1.**
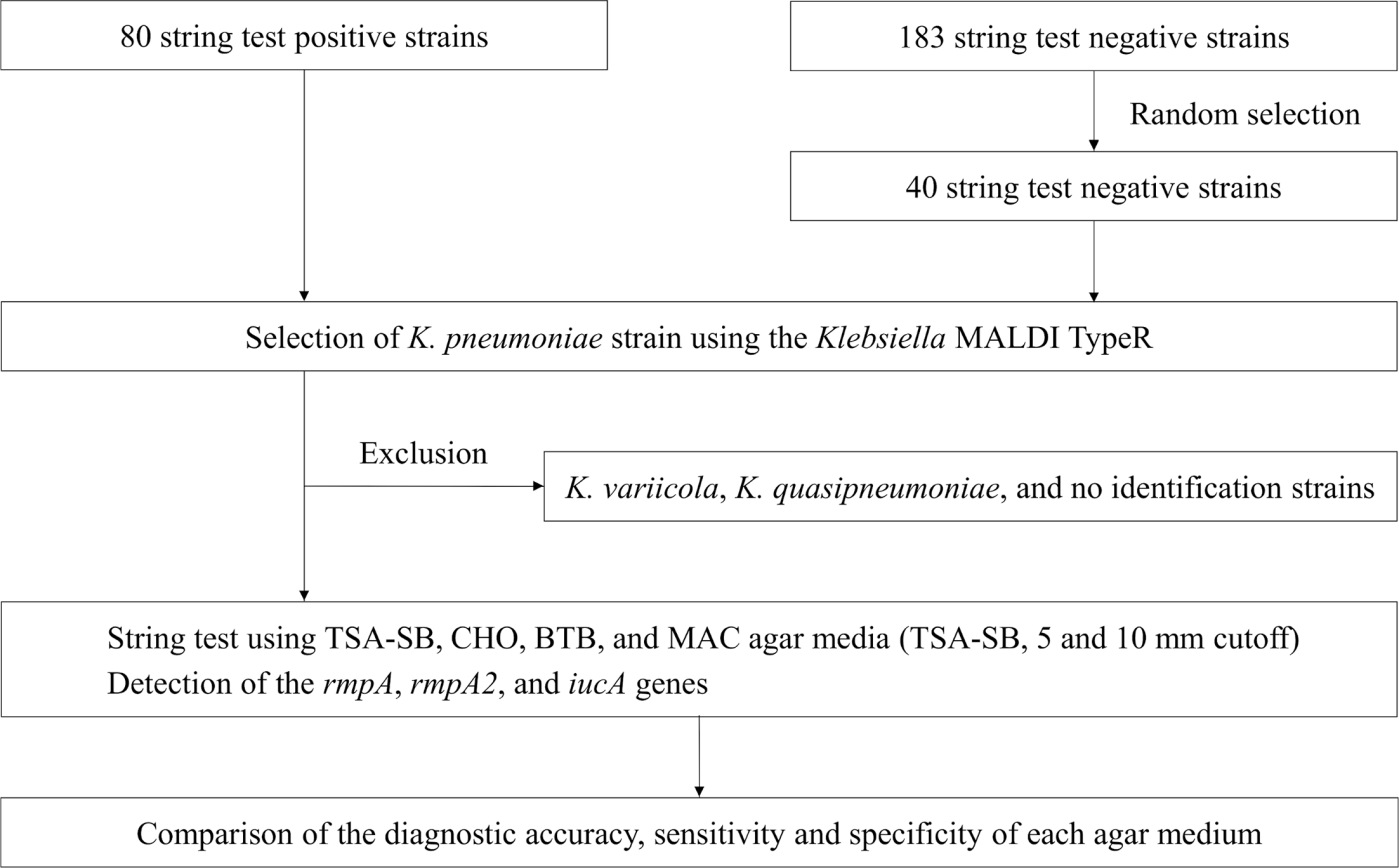
Flowchart of the strains used, string test conditions and evaluation factors. Each factor was evaluated based on its concordance with the virulence genes. BTB, BD BBL-modified Drigalski’s medium; CHO, chocolate II agar medium; MAC, BD BBL MacConkey agar medium; TSA-SB, trypticase soy agar with 5 % sheep blood.

We isolated *Klebsiella* strains from patients at Kameda Medical Center between February 2020 and August 2022. These strains were identified as *K. pneumoniae* or *Klebsiella variicola* through routine testing using matrix-assisted laser desorption ionization-time of flight mass spectrometry (MALDI-TOF MS). Only the first strain was used for the cases wherein multiple strains were isolated from the same patient. Among the isolated strains, 80 and 183 exhibited positive and negative results in the string test, respectively (Fig. 1). The positive strains were string positive with either 5 % sheep blood, chocolate or Drigalski’s medium in routine testing. Positive strains were isolated from various specimens such as blood, urine and sputum, whereas the negative strains were exclusively isolated from blood, of which 40 strains were randomly selected. Consequently, 80 positive and 40 negative strains were selected for the subsequent identification process.

### Identification of *K. pneumoniae* strains

Using *Klebsiella* MALDI TypeR, a web-based tool for *Klebsiella* identification [13], we identified 80 positive and 40 negative string test strains. For this step, the mass spectra were collected from the strain and analysed using the web-based interface. The mass spectra were collected using a MALDI Biotyper system (Microflex LT/SH and FlexControl version 3.4, Bruker Daltonics GmbH, Bremen, Germany) and subsequently uploaded to the web-based tool. The *Klebsiella* strains were classified as *K. pneumoniae*, *K. variicola* and *Klebsiella quasipneumoniae. K. variicola*, *K. quasipneumoniae* and any unidentified strains were excluded from subsequent testing. For the *K. pneumoniae* strains, we collected patient and specimen data from medical records and laboratory systems. Patient data encompassed age, sex (male or female) and patient status (inpatient or outpatient). Patients who were hospitalized at the time of sample collection were considered as inpatients, whereas all the other patients were considered as outpatients.

### Classification of hypervirulent and classical *K. pneumoniae*

Previous studies have suggested that the *rmpA*, *rmpA2* and *iucA* genes are useful markers for hvKp [6]. After identifying these genes, we classified the strains as hvKp or cKp based on their presence. hvKp was defined as a strain positive for one or more of the *rmpA*, *rmpA2* and *iucA* genes, whereas cKp was defined as a strain negative for all the genes.

The *rmpA*, *rmpA2* and *iucA* genes were detected using a previously reported PCR method [6]. DNA templates were prepared using the Cica GeneusR DNA extraction reagent (Kanto Chemical, Tokyo, Japan) following the manufacturer’s instructions. Forward and reverse primers were used as previously described [6]. The PCR reaction mixture contained 25 µl of Premix Ex Taq Hot Start Version (Takara, Tokyo, Japan), 2 µl of forward primer, 2 µl of reverse primer, 2 µl of template DNA and 19 µl of distilled water. PCR amplification was performed using GeneAtlas Type G (Astec, Fukuoka, Japan). PCR conditions were as follows: 95 °C for 2 min, [95 °C for 30 s, annealing temperature (59 °C for *iucA* and 50 °C for *rmpA* and *rmpA2*) for 30 s and 72 °C for extension (50 s for *iucA* and 40 s for *rmpA* and *rmpA2*)] for 25 cycles and 72 °C for 10 min. The amplification products (332 bp for *rmpA*, 430 bp for *rmpA2* and 583 bp for *iucA*) were visualized by electrophoresis using a 2.0 % agarose gel with tris-acetate-ethylenediaminetetraacetic acid buffer at 100 V for 35 min, followed by staining with 1.0 % ethidium bromide.

### String test

String tests were performed, as previously described by Fang *et al*. [5]. The strains were inoculated onto agar media and aerobically incubated at 35 °C for 20–24 h. The colonies on the agar media were pulled up using a 10-µl plastic loop, and the viscous string formation was observed (Supplementary Video 1). The string test was conducted at five different locations on the agar media. A positive string test result was defined as a viscous string formation of >5 mm at one or more spots. Two laboratory scientists performed string tests. In cases of discordant results between the two laboratory scientists, the third laboratory scientist performed the string test, and these results were adopted.

The following agar media were used: trypticase soy agar supplemented with 5 % sheep blood (TSA-SB; Becton, Dickinson and Company, Franklin Lakes, NJ, USA), chocolate II agar medium (CHO; Becton, Dickinson and Company), BD BBL-modified Drigalski’s medium (BTB; Becton, Dickinson and Company) and BD BBL MacConkey agar medium (MAC; Becton, Dickinson and Company). BTB and MAC were prepared according to the manufacturer’s instructions. The TSA-SB was additionally evaluated for the string test with a 10-mm cutoff.

### Statistical analysis

Statistical analysis and summary of each *Klebsiella* group were performed using EZR version 1.54 [14]. The statistical significance was considered at a *P*-value ˂ 0.05. The proportion of positive string tests under each test condition and the *Klebsiella* groups (hvKp and cKp) was evaluated using Fisher’s exact test. The comparison of proportions between groups with one to two virulence genes and those with three virulence genes was performed using Fisher’s exact test. The sensitivity, specificity and diagnostic accuracy of the string test under each condition were calculated. Each factor was calculated as follows. The sensitivity was determined by dividing the number of positive string test results for hvKp by the total number of hvKp strains. The specificity was calculated by dividing the number of negative string test results for cKp by the total number of cKp strains. The diagnostic accuracy was determined by dividing the number of strains for which the string test correctly identified the *Klebsiella* group by the total number of strains. For example, if there are 100 total strains and the string test correctly identified 85 of them, the diagnostic accuracy would be 85 divided by 100, resulting in a diagnostic accuracy of 0.85. For TSA-SB, we compared the number of positive string test results between hvKp and cKp using the Mann–Whitney *U* test. The average positive number determined by two or three clinical laboratory scientists was used for the Mann–Whitney *U* test. The correlation coefficients between the positive string test results using TSA-SB and the presence of specific virulence genes (*rmpA*, *rmpA2* and *iucA*) were calculated.

## Results

### String test strain characteristics

Of the initial 120 strains, 99 (72 positive and 27 negative string test strains) were identified as *K. pneumoniae*. The remaining 21 strains (8 positive and 13 negative string test strains) were identified as non-*K. pneumoniae* and excluded from subsequent analysis. The marker genes led to the categorization of the 99 *K*. *pneumoniae* strains, with 57 (58 %) identified as hvKp and 42 (42 %) as cKp.

**Fig. 2.**
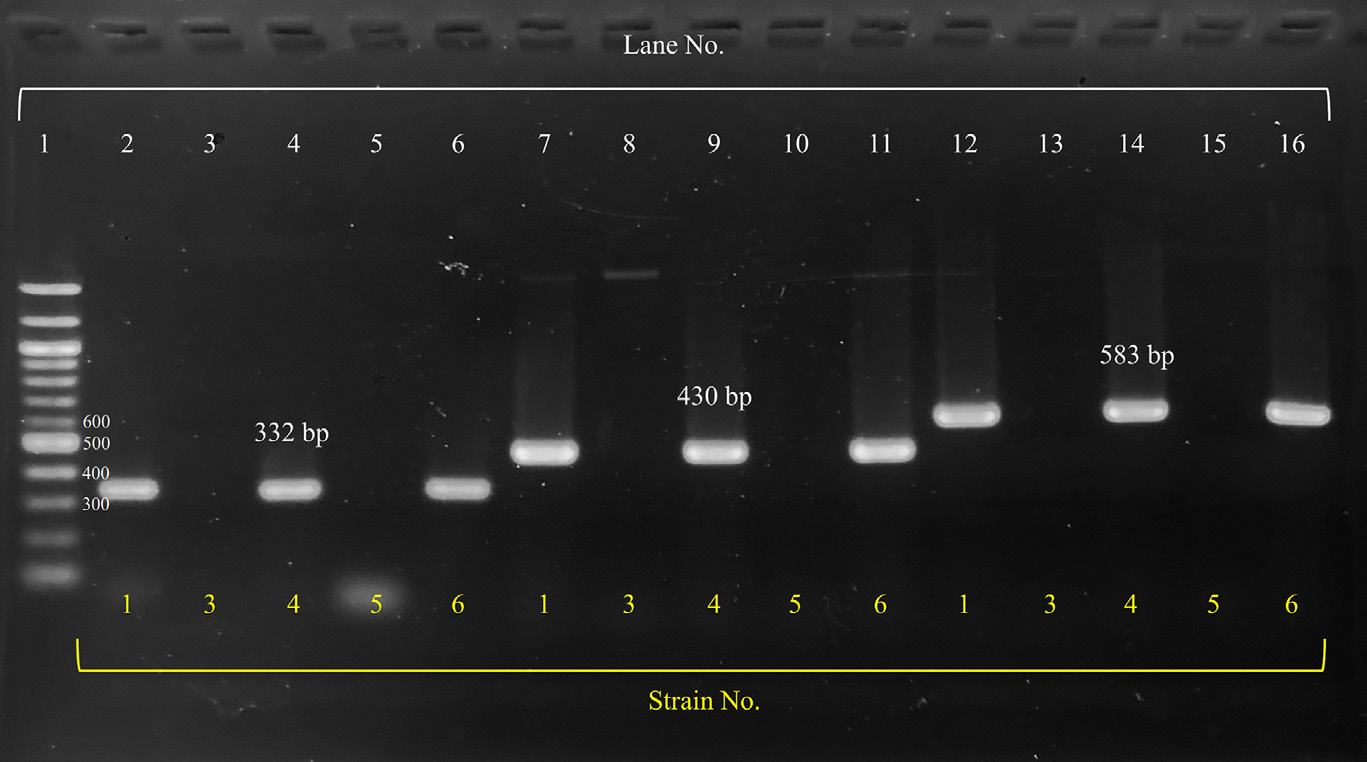
Agarose gel electrophoresis for the detection of *rmpA*, *rmpA2* and *iucA* genes. Lane 1 contains the 100 bp ladder for size reference. Lanes 2–6 correspond to strains 1, 3, 4, 5 and 6, respectively, indicating the presence of the *rmpA* gene (332 bp). Lanes 7–11 represent the same strains mentioned earlier in the same order, showing the *rmpA2* gene (430 bp). Lanes 12–16 also correspond to the same strains in the same order, indicating the presence of the *iucA* gene (583 bp). For each gene, strains 1, 4 and 6 were positive, whereas strains 3 and 5 were negative.

Patient and virulence gene data for each *K. pneumoniae* group are presented in Table 1. The detection of *rmpA*, *rmpA2* and *iucA* genes in *K. pneumoniae* strains was confirmed by agarose gel electrophoresis, as shown in Fig. 2. The median patient age was 72 years [interquartile range (IQR): 61, 80) for hvKp and 77 years (IQR: 69, 85) for cKp. The proportion of females was 40 % (23/57) for hvKp and 43 % (18/42) for cKp. The proportion of inpatients was 49 % (28/57) for hvKp and 43 % (18/42) for cKp. Among the specimens, blood (23 hvKp and 29 cKp) was the most common source, followed by respiratory (23 hvKp and 4 cKp). For the hvKp strains, the detection rates of marker genes were 97 % (55/57) for *rmpA*, 81 % (46/57) for *rmpA2* and 90 % (51/57) for *iucA*.

**Table 1.** Characteristics of hvKp and cKp strains

Characteristics	hvKp (*n*=57)	cKp (*n*=42)
Age, median, years (IQR)	72 (61, 80)	77 (69, 85)
Sex, female, no. (%)	23 (40)	18 (43)
Inpatient, no. (%)	28 (49)	18 (43)
Specimen, no. (%)		
Abscess	2 (4)	0 (0)
Bile	1 (2)	0 (0)
Blood	23 (40)	29 (69)
Respiratory	23 (40)	4 (10)
Urine	5 (9)	9 (21)
Other	3 (5)	0 (0)
Virulence gene, no. (%)		
* rmpA*	55 (97)	0 (0)
* rmpA2*	46 (81)	0 (0)
* iucA*	51 (90)	0 (0)

### Results of the string test for each test condition

String test results showed a significantly higher proportion of positive strains in the hvKp group (58–83 %) when compared to that in the cKp group (24–41%) (*P*-value range: 0.01 to <0.001, Table 2). The highest proportion of positive strains was observed for BTB with 65 % (64/99), followed by TSA-SB with 59 % (58/99). In the hvKp group, the highest proportion of positive strains was observed with TSA-SB at 83 % (47/57) and BTB at 83 % (47/57). In the cKp group, the lowest proportion of positive strains was observed for CHO at 24 % (10/42) and TSA-SB at 26 % (11/42). Of the 42 cKp strains, 17 strains (41 %) were positive for BTB and 16 strains (38 %) were positive for MAC. A moderate correlation was observed between the positive string test results using TSA-SB and the presence of specific virulence genes. The correlation coefficients were 0.57 [95 % confidence interval (CI), 0.40–0.73] for *rmpA*, 0.48 (0.31–0.65) for *rmpA2* and 0.53 (0.37–0.70) for the *iucA* gene.

**Table 2.** Proportion of string test positive strains in hypervirulent and classical *K. pneumoniae*

Test condition	No. of positive strains (%)		***P*-value**
	hvKp (*n*=57)	cKp (*n*=42)	
5 -mm cutoff			
TSA-SB	47 (83)	11 (26)	<0.001
CHO	42 (74)	10 (24)	<0.001
BTB	47 (83)	17 (41)	<0.001
MAC	37 (65)	16 (38)	0.01
10-mm cutoff			
TSA-SB	33 (58)	11 (26)	0.002

Table 3 shows the number of positive string test strains in the groups with one or two virulence genes and those with three virulence genes. For TSA-SB, the proportion of positive strains was higher in the three virulence genes group (85 %) than in the one or two virulence gene groups (73 %). This trend was also observed for BTB. For CHO, the proportion of positive strains in the three virulence gene groups (74 %) was comparable to that in the one or two virulence gene groups (73 %). A similar trend was observed for MAC. Among all media, no significant differences in the proportion of positive strains were observed between the one or two virulence gene groups and the three virulence gene groups (*P*-value range: 0.1 to 1).

**Table 3.** Comparison of the proportion of positive string tests for groups with one to two and three virulence genes

Test condition	No. of positive strains (%)		*P*-value
	Three genes (*n*=46)	One or two genes (*n*=11)	
5-mm cutoff			
TSA-SB	39 (85)	8 (73)	0.4
CHO	34 (74)	8 (73)	1
BTB	40 (87)	7 (64)	0.1
MAC	30 (65)	7 (64)	1
10-mm cutoff			
TSA-SB	27 (59)	6 (55)	1

### Detection performance of the string test for each test condition

The detection performance of the string test using different agar media is presented in Table 4. The highest sensitivity rates were observed for TSA-SB and BTB, both at 83 % (95 % CI, 70–91). CHO with 76 % (61–88) yielded the highest specificity, followed by TSA-SB with 74 % (58–86). For the diagnostic accuracy, TSA-SB with 0.79 (95 % CI, 0.69–0.86) exhibited the highest value, followed by CHO with 0.75 (0.65–0.83), BTB with 0.73 (0.63–0.81) and MAC with 0.64 (0.53–0.73).

**Table 4.** Diagnostic accuracy, sensitivity and specificity of the string test

Test condition	Detection performance (95 % CI)		
	Accuracy	Sensitivity (%)	Specificity (%)
5-mm cutoff			
TSA-SB	0.79 (0.69–0.86)	83 (70–91)	74 (58–86)
CHO	0.75 (0.65–0.83)	74 (60–85)	76 (61–88)
BTB	0.73 (0.63–0.81)	83 (70–91)	60 (43–74)
MAC	0.64 (0.53–0.73)	65 (51–77)	62 (46–76)
10-mm cutoff			
TSA-SB	0.65 (0.54–0.74)	58 (44–71)	74 (58–86)

### Comparison between 5- and 10-mm cutoff values

Of the 47 positive string test strains observed using TSA-SB, 33 (70 %) were positive at the 10-mm cutoff. The sensitivity of the 5-mm cutoff (83 %; 95 % CI, 70–91) exceeded that of the 10-mm cutoff (58 %, 44–71). The specificity was similar at 74 % for both cutoffs. Upon comparing the 5- and 10-mm cutoffs, the diagnostic accuracy of the 5-mm cutoff (0.79; 95 % CI, 0.69–0.86) was found to be higher than that of the 10-mm cutoff (0.65, 0.54–0.74).

### Number of positive string test results

The median number of positive string test results was five (range, 0.7–5) for hvKp and five (range, 2.5–5) for cKp. Among the hvKp strains, 68 % (32/47) were positive five times, whereas the remaining 32 % (15/47) were positive less than five times. Of the cKp strains, 73 % (8/11) were positive five times, and the remaining 27 % (3/11) were positive less than five times. The number of positive string test results for hvKp was not statistically significantly different from that of cKp (*P*-value=0.9).

## Discussion

In this study, we evaluated the impact of agar media and cutoffs on string test results. String test results obtained using various agar media were associated with the *Klebsiella* groups. Among the evaluated agar media, TSA-SB with a 5-mm cutoff exhibited the highest diagnostic accuracy.

Since the introduction of the string test by Fang *et al.* [5], this test has become a widely used tool in *Klebsiella* studies. Several studies have employed different agar media [7,10]. Our results indicate that TSA-SB was the most appropriate agar medium for string tests. Compared to the CHO, BTB and MAC string tests, the TSA-SB string test showed higher accuracy. TSA-SB exhibited an accuracy, sensitivity and specificity of 0.79, 83 % and 74 %, respectively, for identifying hvKp. Given its common usage in clinical laboratory testing [15], TSA-SB is a practical agar medium choice for performing string tests.

The BTB string test yielded the highest strain number of positive string test results. However, owing to its low specificity (60 %), the use of BTB may lead to the overdiagnosis of hvKp. In contrast, the CHO string test showed higher specificity (76%) but lower sensitivity (74 %) than the other agar media, which may lead to an underestimation of hvKp. However, this performance may have limited the applicability. Sensitivity and specificity values for the same test may vary depending on the target population. For example, in an environment with a high prevalence of cKp and a very low prevalence of hvKp, the CHO string test with the higher specificity may result in a higher overall accuracy when compared to the TSA-SB string test. Given the factors that contribute to the detection performance of each agar medium, one potential contributor could be the components of each agar medium. For instance, the previous study has demonstrated that glucose can stimulate capsular polysaccharide biosynthesis in *K. pneumoniae* [16]. The components of the agar medium, such as glucose, lactose and peptone, may have affected the string test results.

Previous studies have reported variations in string test results depending on cultivation parameters [17]. Neumann *et al.* evaluated the influence of culture conditions on string test results, and the results indicate a correlation between prolonged string length and hypervirulence genes under anaerobic growth on Mueller–Hinton agar [17]. However, we did not test the anaerobic culture conditions on Mueller–Hinton agar as we focused on string testing for agar and culture conditions in clinical practice, wherein aerobic culture is typically used. The different culture conditions and media used in the two studies make direct comparison challenging. However, a more robust analysis of the diagnostic performance of the string test under different media conditions may be possible due to the larger number of hvKp strains in our study (47 compared to 9 in the study by Neumann *et al.*). Additionally, previous reports have shown that the string test result is temperature dependent [18]. We evaluated the string test at 35–37 °C to evaluate its use in medical practice, but the temperature dependence of hypermucoviscosity can be noted.

Our results suggested a 5-mm cutoff as appropriate for string tests. This aligns with the common practice of employing the 5-mm cutoff, as reported by Fang *et al.* [5]. However, several studies have used cutoffs of 10 mm or more [11,12]. Our study demonstrated that the diagnostic accuracy of the 5-mm cutoff (0.79) was superior to that of the 10-mm cutoff (0.65). Additionally, the number of positive string test results was similar between hvKp and cKp, indicating that the number of positive results cannot distinguish hvKp from cKp.

The string test can be performed simply by lifting a colony on agar media, making it easy to implement in routine testing. However, hypervirulence and hypermucoviscosity are two different phenomena [7]. According to a study by Wu *et al.*, 7 % of hvKp strains were string test negative and 4 % of cKp strains were *rmpA* gene positive [19]. This discrepancy between hypervirulence and hypermucoviscosity has also been reported in other studies [6,20]. In our study, 26 % of cKp strains had a positive string test result, which is consistent with previous reports that hypervirulence and hypermucoviscosity are two different phenomena. The string test is easy to use, but hypervirulence and hypermucoviscosity are two different phenomena, and this test should be used considering its limitations.

This study had certain limitations. The *Klebsiella* groups were defined using *rmpA*, *rmpA2* and *iucA* genes, which exhibit a high estimated diagnostic accuracy of 0.95–0.96 [6]. However, these genes do not completely differentiate between hvKp and cKp. Therefore, some strains might have been misclassified as belonging to the *Klebsiella* group. Additionally, only four types of agar media were evaluated in this study. The components of the agar media can vary among manufacturers; therefore, our results may not apply to string tests performed using agar media from other manufacturers. This study did not evaluate sequence typing, *in vivo* assays such as mouse infection models, hvKp-associated capsular types or clinical aspects. Moreover, this study did not evaluate the relationship between these factors and string test results. Therefore, the results of this study may not be applicable to *K. pneumoniae* populations collected in different regions or facilities. Further research is needed to determine the effect of these factors on string test results.

## Conclusions

Our findings indicate that the type of agar medium used affected the string test results. The diagnostic accuracy of TSA-SB with a 5-mm cutoff was superior to that of other media. In conclusion, optimizing string test conditions can enhance the diagnostic accuracy for hvKp.

## Abbreviations

BTB: BD BBL-modified Drigalski's medium
CHO: chocolate II agar medium
CI: confidence interval
cKp: classical *Klebsiella pneumoniae*
hvKp: hypervirulent Klebsiella pneumoniae
IQR: interquartile range
MAC: BD BBL MacConkey agar medium
MALDI-TOF MS: matrix-assisted laser desorption ionization-time of flight mass spectrometry
TSA-SB: trypticase soy agar with 5% sheep blood
